# Enhancing regional disease burden estimates: insights from the comparison of Global Burden of Disease and China’s notifiable infectious diseases data with policy implications (2010–2020)

**DOI:** 10.1186/s40249-025-01351-3

**Published:** 2025-08-06

**Authors:** Zi-Yu Zhao, Jiao-Jiao Li, Han-Qi Ouyang, Wei-Hao Li, Sheng-Kai Huang, Okugbe Ebiotubo Ohore, Lu Wang, Jürg Utzinger, Guo-Jing Yang

**Affiliations:** 1 School of Public Health, Hainan Medical University, Haikou, Hainan People’s Republic of China; 2 NHC Key Laboratory of Tropical Disease Control, School of Tropical Medicine, Hainan Medical University, Haikou, Hainan 571199 People’s Republic of China; 3 Department of Infectious Diseases, the First Affiliated Hospital of Hainan Medical University, Haikou, 570102 Hainan People’s Republic of China; 4 Chinese Center for Disease Control and Prevention, Beijing, 100050 People’s Republic of China; 5 Swiss Tropical and Public Health Institute (Swiss TPH), University of Basel, Basel, Switzerland

**Keywords:** Notifiable infectious diseases, Disability-adjusted life years, Global Burden of Disease study 2021, China, Control policy

## Abstract

**Background:**

The Global Burden of Disease (GBD) study offers influential Disability-Adjusted Life Years (DALYs) estimates for various diseases. However, discrepancies with national surveillance data raise concerns about accuracy. This study aims to promote the deep integration of the GBD model with localized data and facilitate the development of region-specific models.

**Methods:**

Data for 14 notifiable infectious diseases (NIDs), grouped into intestinal infectious diseases, respiratory infectious diseases, and sexually transmitted and blood-borne infections, were obtained from the Data-center of China Public Health Science. DALYs based on national surveillance data (2010–2020) were calculated using DALY formulas, and discrepancies with GBD estimates were quantified through ratio comparisons. A historical timeline map highlighted key infectious disease control policies and certified disease elimination events in China.

**Results:**

National surveillance data show a decrease in DALYs for 14 NIDs in China, from 6,529,124.62 person-years in 2010 to 6,326,497.18 person-years in 2020. Among them, sexually transmitted and blood-borne infections have the highest burden, with 78% of DALYs attributed to hepatitis B (4,864,028.29 person-years). Respiratory infectious diseases follow, with 99% of DALYs from TB (394,927.70 person-years). Intestinal infectious diseases have the relative lightest burden, with 45% of DALYs from hepatitis E (496.49 person-years). Over 11 years, 9 of the 14 NIDs showed a downward trend. Comparisons reveal that DALYs based on national surveillance data are lower than GBD 2021 estimates.

**Conclusions:**

Considerable differences exist between the GBD estimates and national surveillance data regarding the burden of 14 NIDs in China. Therefore, strengthening national reporting systems and integrating localized data with the GBD model is essential for more accurate disease burden assessments and effective response strategies. Despite significant progress in infectious disease control, China still faces substantial challenges in domestic disease elimination.

**Graphical Abstract:**

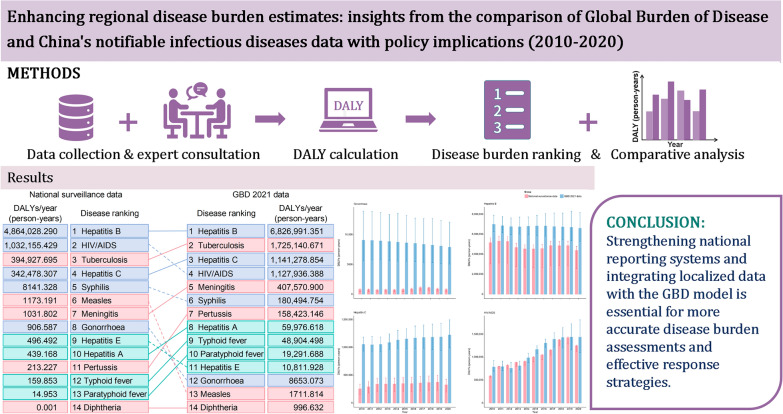

**Supplementary Information:**

The online version contains supplementary material available at 10.1186/s40249-025-01351-3.

## Background

Infectious diseases remain one of the most urgent global health challenges, particularly in low- and middle-income countries [1]. With limited global resources, there is a pressing need for comprehensive metrics to guide public health priorities and resource allocation, balancing emergency response with long-term planning [2]. Disability-adjusted life years (DALYs) have emerged as a key measure for quantifying the overall burden of disease, offering critical insights into the impact of infectious diseases [3]. Since its inception in 1990, the Global Burden of Disease (GBD) study has provided a foundational framework for burden of disease research at regional, national, and global levels [3]. GBD data have been widely used to inform global health policies, contributing to landmark findings in *The Lancet* on mortality, disease rankings, and risk factors [4, 5]. However, existing studies suggest that GBD estimates may significantly deviate from reported data on infectious diseases [6]. The GBD framework relies heavily on model-based estimates, which are built on assumptions and indirect data. This reliance may introduce substantial inaccuracies, particularly in countries with complex disease spectra, limiting the utility of GBD data in specific contexts.

In recent years, a growing number of studies based on the GBD data have been published, highlighting the field's heavy reliance on these data. To examine the current state of infectious disease burden research in China, we used keywords such as “infectious diseases”, “disease burden”, “DALY”, and “China” to search for publications from 2019 to 2024 in the China National Knowledge Infrastructure (https://www.cnki.net/) database before this study. The findings reveal that 68% (38/56) of studies on the burden of infectious diseases in China relied on GBD data. This heavy reliance on GBD data may not fully reflect the real-world disease burden at the regional level, potentially limiting its applicability for regional decision-making. Despite 32% (18/56) of the studies used local surveillance data to estimate diseases burden, they primarily focus on single diseases or provincial-level assessments, lacking comprehensive nationwide analyses. Comprehensive evaluations of the burden of infectious diseases are crucial for formulating national public health policies, allocating resources, and planning intervention measures. Integrating local surveillance data can enhance the accuracy of burden estimates and address the regional details that global models may overlook.

China, the most populous country in the world, faces unique challenges in infectious disease prevention and control. Over the past decade, disease surveillance in China has improved significantly, driven by the National Notifiable Infectious Disease Reporting Information System (NIDRIS), which provides real-time monitoring of 41 notifiable infectious diseases (NIDs) [7] and offers incidence and mortality data from 2004 to 2020. However, due to incomplete diagnostic criteria and reporting mechanisms, the initial data quality was low. Based on recommendations from experts at the Chinese Center for Disease Control and Prevention (China CDC), data from 2010 onward are advised to ensure quality and accuracy. Existing studies have yet to use DALY as a comprehensive metric to quantify the burden of multiple NIDs, making it impossible to identify the diseases with the highest burden. For example, a review of the trends in all NIDs in China from 2010 to 2019 shows that the incidence rates of Class-A and Class-B infectious diseases have been decreasing [8]. Nor have systematic efforts been made to directly compare GBD estimates with China’s surveillance data. These gaps limit the decision-making utility of GBD data within the Chinese context.

To address these gaps, this study uses China as a reference to analyzes the current state of infectious disease burden research and explores how integrating GBD estimates with local surveillance data can enhance evaluation. By utilizing China’s surveillance data (2010–2020), DALYs for 14 NIDs were calculated and compared with GBD 2021 estimates. Within the framework of China’s disease prevention strategy, the study prioritizes disease burden to guide public health resource allocation, policy formulation, and the integration of GBD models with localized data.

## Methods

### Data archive

NIDRIS monitors 41 NIDs, categorized by China CDC into five groups by transmission routes: intestinal infectious diseases, respiratory infectious diseases, natural focal and vector-borne diseases, sexually transmitted and blood-borne infections, and diseases with other transmission routes [9]. Among these, diseases with other transmission routes includes multiple neglected tropical diseases, which have been analysed in previous studies [10]. Specifically, this study focuses on three major categories (intestinal infectious diseases, respiratory infectious diseases, and sexually transmitted and blood-borne infections), encompassing 21 diseases. Diseases lacking data in the GBD 2021 database (e.g., poliomyelitis, dysentery, scarlet fever) or with incomplete data in the NIDRIS (e.g., influenza A (H1N1) and hepatitis D) were excluded from the analysis. Additionally, due to the extremely low incidence of cholera and the absence of reported cases of severe acute respiratory syndrome (SARS) in recent years, along with the absence of data for both in the GBD 2021 database, they were excluded. Ultimately, this study included data from 14 NIDs reported between 2010 and 2020 across 31 provincial-level administrative divisions in China (not included Taiwan, Hong Kong, and Macau), comprising 4 intestinal infectious diseases (hepatitis A, hepatitis E, paratyphoid fever, typhoid fever), 5 respiratory infectious diseases [diphtheria, measles, meningitis, pertussis, tuberculosis (TB)], and 5 sexually transmitted and blood-borne infections [gonorrhoea, hepatitis B, hepatitis C, human immunodeficiency virus/acquired immunodeficiency syndrome (HIV/AIDS), syphilis].

As a globally recognized disease classification and diagnostic standard, the International Classification of Diseases (ICD) codes are used to systematically classify, identify, and record diseases, health conditions, injuries, and causes of death worldwide [11]. The GBD 2021 study mapped ICD codes, for both ICD-9 (1975) [12] and ICD-10 (1994) [13], to the GBD 2021 causes of death and nonfatal causes [14]. Additionally, the coding systems adopted by the China CDC from 2010 to 2020 (GB/T 14396–2001 [15] and GB/T 14396–2016 [16]) align with the ICD-10 standard of the World Health Organization (WHO). Therefore, we reviewed the coding for 14 NIDs across the coding standards published by China and ICD-10 to ensure consistency and comparability. The list of ICD codes mapped to 14 NIDs in the GBD 2021, along with the Classification and Codes of Diseases mapped to the 14 diseases in China, is provided in supplementary table S1.

Data on incident cases and deaths (by age and region) for 14 NIDs (2010–2020) were obtained from the Data-center of China Public Health Science (https://www.phsciencedata.cn/Share/index.jsp). Life expectancy in China during the same period were sourced from the China Statistical Yearbook [17]. Disease durations were determined through expert consultation and literature (see supplementary table S2). Disability weights (DWs) were based on related literature and the Global Health Data Exchange (https://ghdx.healthdata.org/record/ihme-data/gbd-2019-disability-weights), which is an online platform that publishes GBD results. Based on the DWs estimated from the GBD, this study recalculated the DWs according to the proportion of various symptoms of the 14 diseases in China (S2 Table). Estimated DALYs for the 14 NIDs in the same period were extracted from the GBD 2021 database (https://vizhub.healthdata.org/gbd-results/) for comparative analysis.

### Data analysis

#### DALY calculation

To ensure consistency with the methodologies employed in the GBD study, this research utilizes a simplified DALY formula provided by the WHO [18, 19], to calculate the disease burden of NIDs in China (2010–2020). The number of incident cases and deaths were collected to calculate years of life lost (YLLs) and years lived with disability (YLDs), which were then summed to obtain DALYs. The formula is as follows:

1 $$YLL\, = \,N \times L;$$

2 $$YLD\, = \,I \times d \times DW;$$

3 $$DALY\, = \,YLL + YLD;$$

where N represents the number of deaths; and L is standard life expectancy at age of death in China (in years); I is the number of incident cases; d is the average duration of disease (in years); DW is the disability weight. DALYs for hepatitis A, hepatitis E, paratyphoid fever, typhoid fever, diphtheria, measles, meningitis, pertussis, TB, gonorrhoea, hepatitis B, and hepatitis C are calculated directly using the above formula. For HIV/AIDS and syphilis, the DALY values for each disease stage are calculated separately, and then summed to obtain the total DALYs for each disease. Except for the DW for pertussis, which is referenced from GBD 2019, the DWs for the remaining 13 diseases are sourced from GBD 2021.

The 95% uncertainty intervals for the DALYs associated with each disease were estimated using the Monte Carlo Simulation method, with 10,000 iterations [20]. In the simulation process, triangular distributions were applied to the relevant parameters to account for the inherent uncertainty in the data.

#### Visualization of DALY trends

DALYs for 14 NIDs in China (2010–2020) were calculated based on reported data and visualized using various graphical methods. Line charts were used to illustrate temporal changes in the overall disease burden, while bubble charts highlighted the relative contributions of each disease within the three major categories. Pie charts were employed to depict the proportional burden of each disease category, and a heatmap was created to illustrate the annual trends for each disease over the 11 year period.

#### Disease burden ranking

The 14 NIDs were ranked based on their 11 year average DALYs calculated from national surveillance data to identify the diseases contributing most significantly to the overall burden. These rankings were then compared with those derived from the GBD 2021 estimates to highlight potential discrepancies in disease prioritization between national surveillance data and modeled estimates.

#### Comparison of national surveillance data and GBD 2021 estimates

Discrepancies between the national surveillance data and GBD 2021 estimates were quantified by calculating the ratios of the 11-year average DALYs from the two sources.

### Prevention and control policies for NIDs in China

Relevant prevention and control policies, guidelines, and achievements for NIDs in China were identified through searches using keywords such as “notifiable infectious diseases,” “immunize,” “prevention and control,” “diagnostic criteria,” “surveillance,” “expert consensus,” and “eliminate.” The search covered official websites of various governmental departments, including the China CDC, the National Health Commission of the People’s Republic of China (http://www.nhc.gov.cn/), the National Development and Reform Commission (https://www.ndrc.gov.cn/), and the State Council Policy Document Library (https://www.gov.cn/zhengce/zhengcewenjianku/index.htm). These platforms provided officially published policies, guidelines, and documents related to infectious disease control (in Chinese). Additionally, milestone achievements in infectious disease control were retrieved from CNKI and WHO-certified reports. Based on this information, a comprehensive historical timeline map was constructed, integrating key policies, guidelines, and certified disease elimination events.

Data extraction and integration were executed using Microsoft Excel 2021 (Microsoft, Redmond, USA), with all analyzes and visualizations conducted using R version 4.4.0 (Lucent Technologies, Jasmine Mountain, USA).

## Results

### DALYs trend of 14 NIDs calculated by national surveillance data

**Fig. 1:**
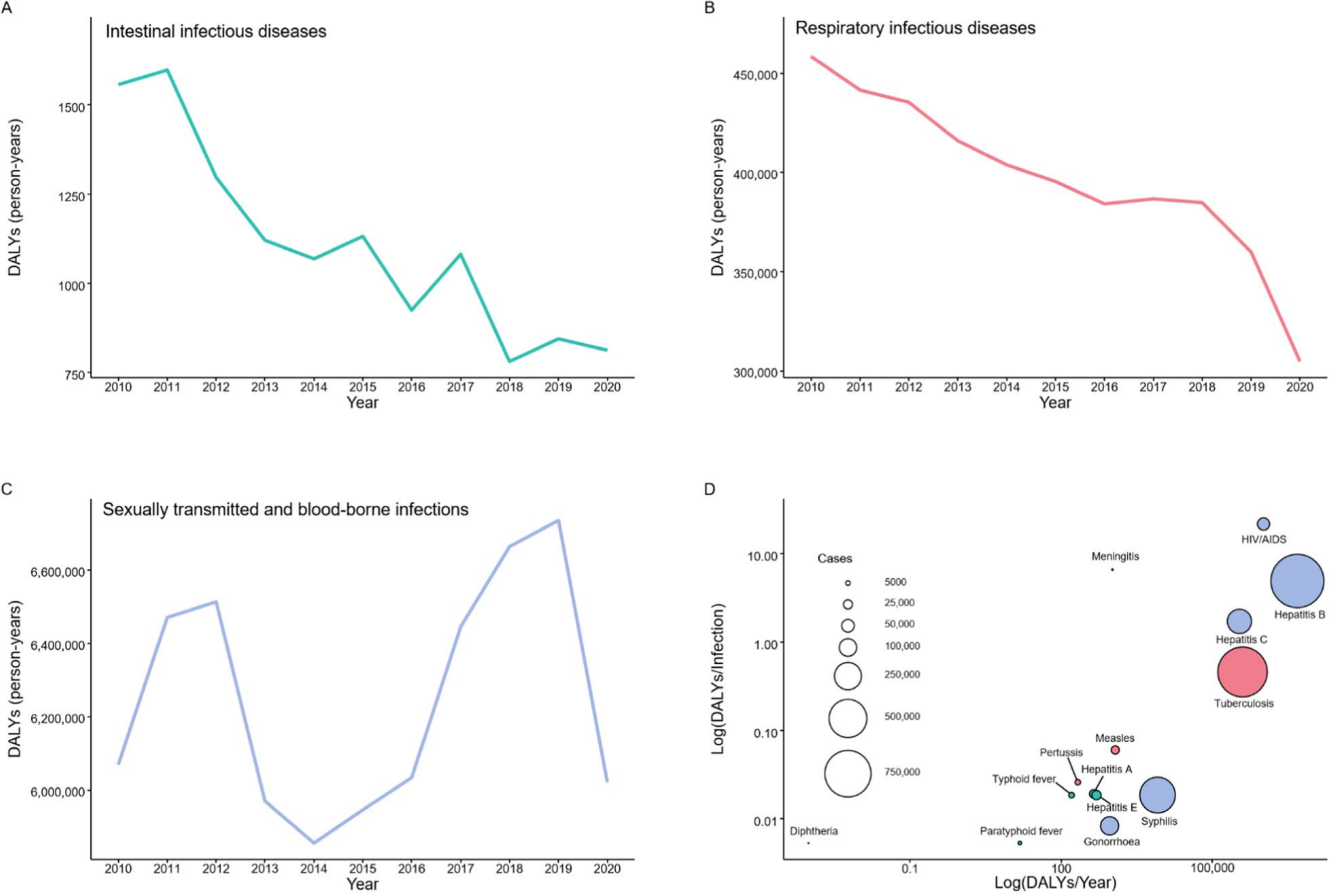
Trends in total disability-adjusted life years (DALYs) of 14 notifiable infectious diseases (NIDs) in China (2010–2020) calculated by national surveillance data. **A** Total DALYs of four intestinal infectious diseases, **B** total DALYs of five respiratory infectious diseases, **C** total DALYs of five sexually transmitted and blood-borne infections, and **D** ranking of 14 NIDs by disease burden at the population/individual level in China from 2010 to 2020. The x-axis is the total DALYs per year, and the y-axis is the DALYs per infection. The area of each bubble is proportional to the 11-year average number of cases. Both axes are on a logarithmic scale. Green represents four intestinal infectious diseases, red represents five respiratory infectious diseases, and purple represents five sexually transmitted and blood-borne infections

The total DALYs for 14 NIDs in China exhibited a slight overall decline, decreasing by 3% (202,627.43 person-years) from 6,529,124.62 in 2010 to 6,326,497.18 in 2020. Among the three major categories of infectious diseases, sexually transmitted and blood-borne infections carried the highest burden, followed by respiratory infectious diseases, while intestinal infectious diseases had a relatively lighter burden. Specifically, DALYs for intestinal infectious diseases decreased by 744.57 person-years, from 1556.7 in 2010 to 812.18 in 2020 (Fig. 1A). For respiratory infectious diseases, DALYs decreased by 153,761.42 person-years, from 458,472.37 in 2010 to 304,710.94 in 2020 (Fig. 1B). In contrast, DALYs for sexually transmitted and blood-borne infections showed an oscillating trend, decreasing by 48,121.44 person-years, from 6,069,095.50 in 2010 to 6,020,974.06 in 2020, with notable peaks observed in 2012 and 2019 (Fig. 1C). Figure 1D shows ranking of 14 NIDs by disease burden at population/individual level. Among the 14 NIDs, HIV/AIDS, hepatitis B, hepatitis C, and TB exhibited high disease burdens at both the population and individual levels. Notably, the bubble sizes for hepatitis B and TB were the largest, signifying the highest average number of cases in China.

**Fig. 2:**
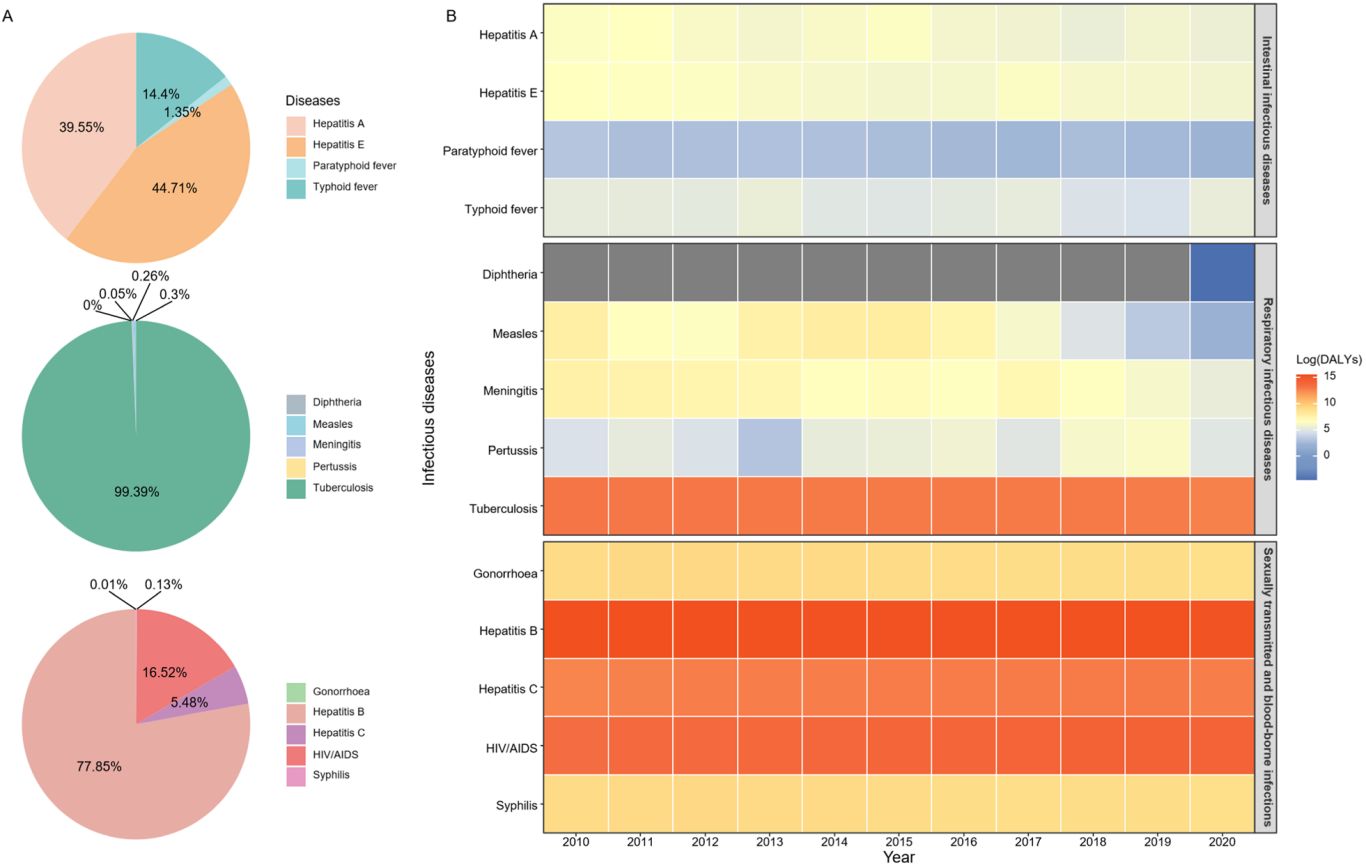
Trend of the Disability-Adjusted Life Years (DALYs) for the 14 notifiable infectious diseases (NIDs) in China (2010–2020) calculated by national surveillance data. **A** The composition of each disease's 11-year average DALYs in the three major categories of diseases included is shown in pie charts. **B** DALYs trend of 14 NIDs in China from 2010 to 2020. The DALYs value is log-transformed for each disease in a specific year, ranging from low (blue) to high burden (red). The grey grids indicates that DALY is zero

Figure 2A shows the disease burden proportions of each disease within the three categories: hepatitis E and hepatitis A accounted for 45% (496.49 person-years) and 40% (439.17 person-years) of intestinal infectious diseases DALYs, respectively; 99% of respiratory infectious diseases DALYs were contributed by TB (4,864,028.29 person-years); while hepatitis B and HIV/AIDS were responsible for 78% (394,927.70 person-years) and 17% (1,032,155.43 person-years) of sexually transmitted and blood-borne infections DALYs, respectively.

Excluding typhoid fever, diphtheria, pertussis, hepatitis C, and HIV/AIDS, the disease burden of the remaining 9 NIDs consistently declined over the years, as depicted in Fig. 2B. Between 2010 and 2020, the DALYs for hepatitis A, hepatitis E, paratyphoid fever, measles, meningitis, TB, gonorrhoea, hepatitis B, and syphilis decreased by 59%, 53%, 62%, 100%, 90%, 33%, 16%, 15%, and 14%, respectively. The DALYs based on national surveillance data for the 14 NIDs and their corresponding GBD estimates are detailed in S3-S5 Tables.

### Disease ranking of 14 NIDs

**Fig. 3:**
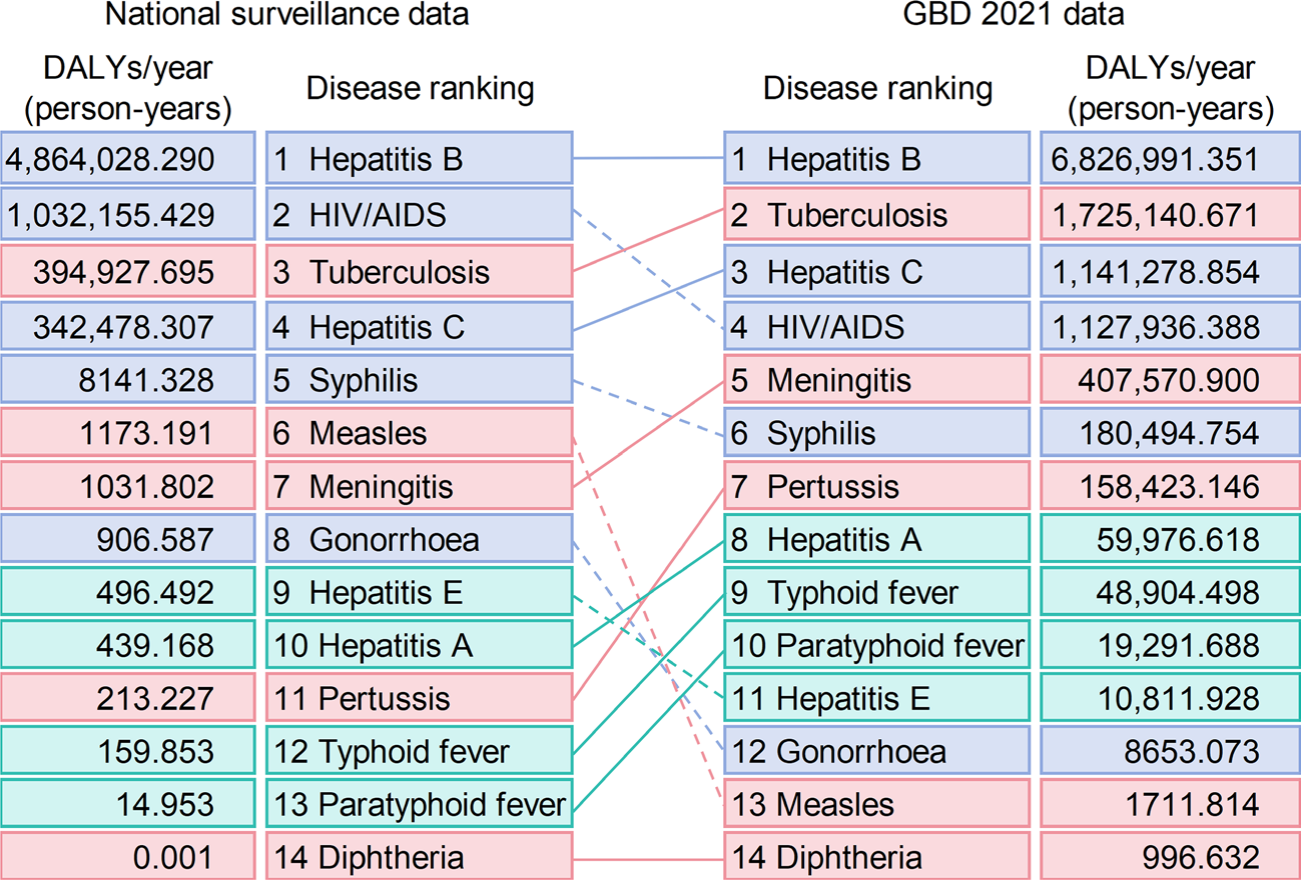
Ranking of 14 notifiable infectious diseases (NIDs) based on the 11-year average Disability-Adjusted Life Years (DALYs). The rankings are derived separately from national surveillance data in China and Global Burden of Disease (GBD) 2021 estimates (2010–2020), and the two rankings are compared. Solid lines are used to connect diseases ranked higher in GBD 2021 estimates compared to DALYs calculated by national surveillance data, while dashed lines are used for diseases ranked higher in DALYs based on national surveillance data compared to GBD 2021 estimates. Green represents the four intestinal infectious diseases, red represents the five respiratory infectious diseases, and purple represents the five sexually transmitted and blood-borne infections

As shown in Fig. 3, based on the rankings of 14 NIDs calculated using the 11-year average DALYs derived from national surveillance data, the leading three diseases are identified as hepatitis B, HIV/AIDS, and TB. In contrast, the GBD 2021 estimates rank the leading three as hepatitis B, TB, and Hepatitis C. A comparison of the rankings of the 14 NIDs derived from the two data sources reveals that only hepatitis B (first) and diphtheria (last) remain consistent in their rankings. Notably, compared to their rankings in national surveillance data, tuberculosis, hepatitis C, meningitis, hepatitis A, pertussis, typhoid fever, and paratyphoid fever have moved up in the GBD 2021 data rankings, while HIV/AIDS, syphilis, measles, gonorrhoea, and hepatitis E have moved down.

### Comparison of DALYs calculated by national surveillance data with GBD 2021 estimates

The discrepancies between the 11-year average of calculated DALYs and the GBD estimates from 2010 to 2020 were quantified using the ratios (see Fig S1). The ratios of GBD estimates to calculated DALYs among the 14 NIDs ranged from 1.09 to 1,035,542.35. For the five diseases with the largest disparities, the GBD 2021 estimates for the 11-year average DALYs of diphtheria, paratyphoid fever, pertussis, meningitis, and typhoid fever are 1,035,542.35, 1,290.18, 742.98, 395.01, and 305.94 times higher than the calculated values, respectively.

**Fig. 4:**
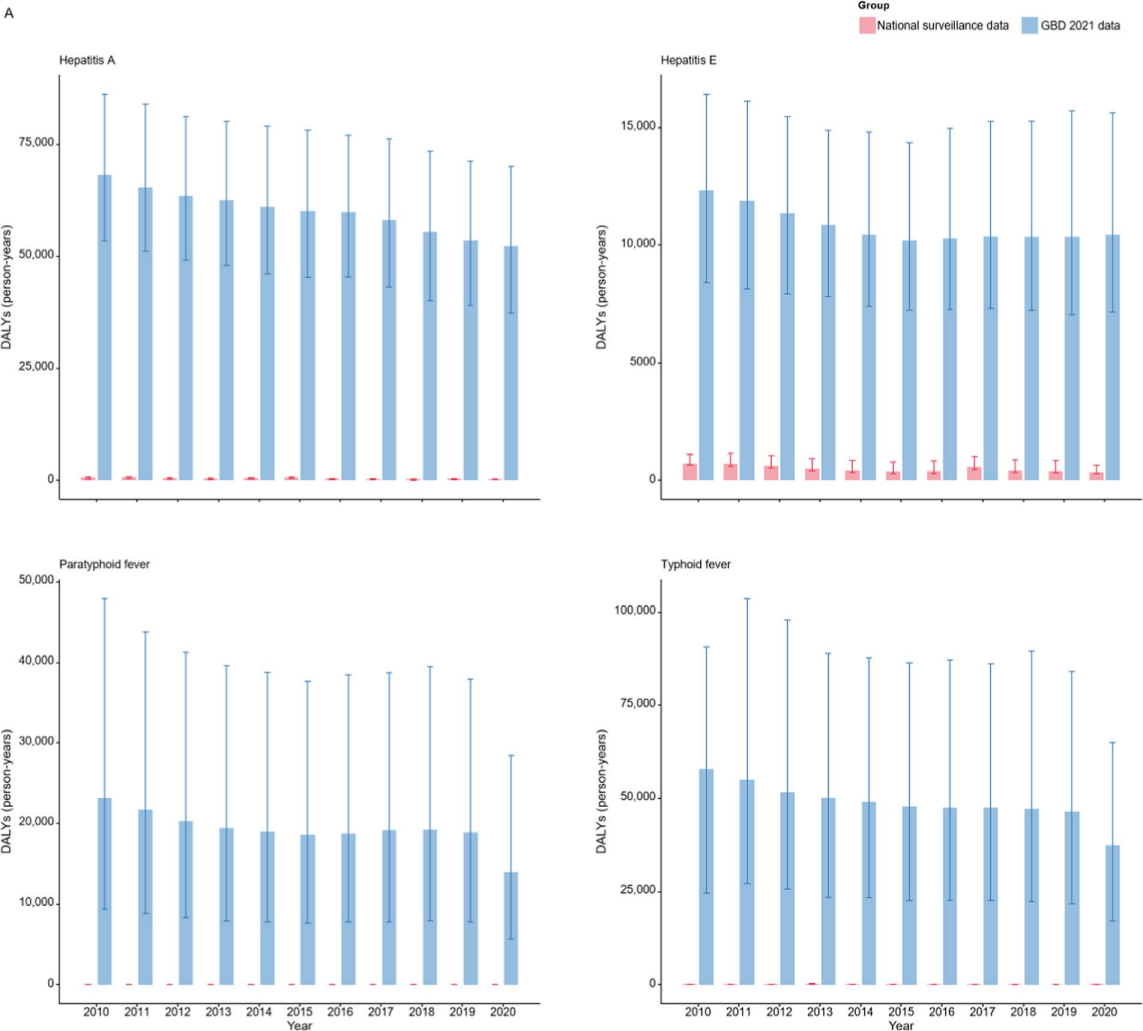

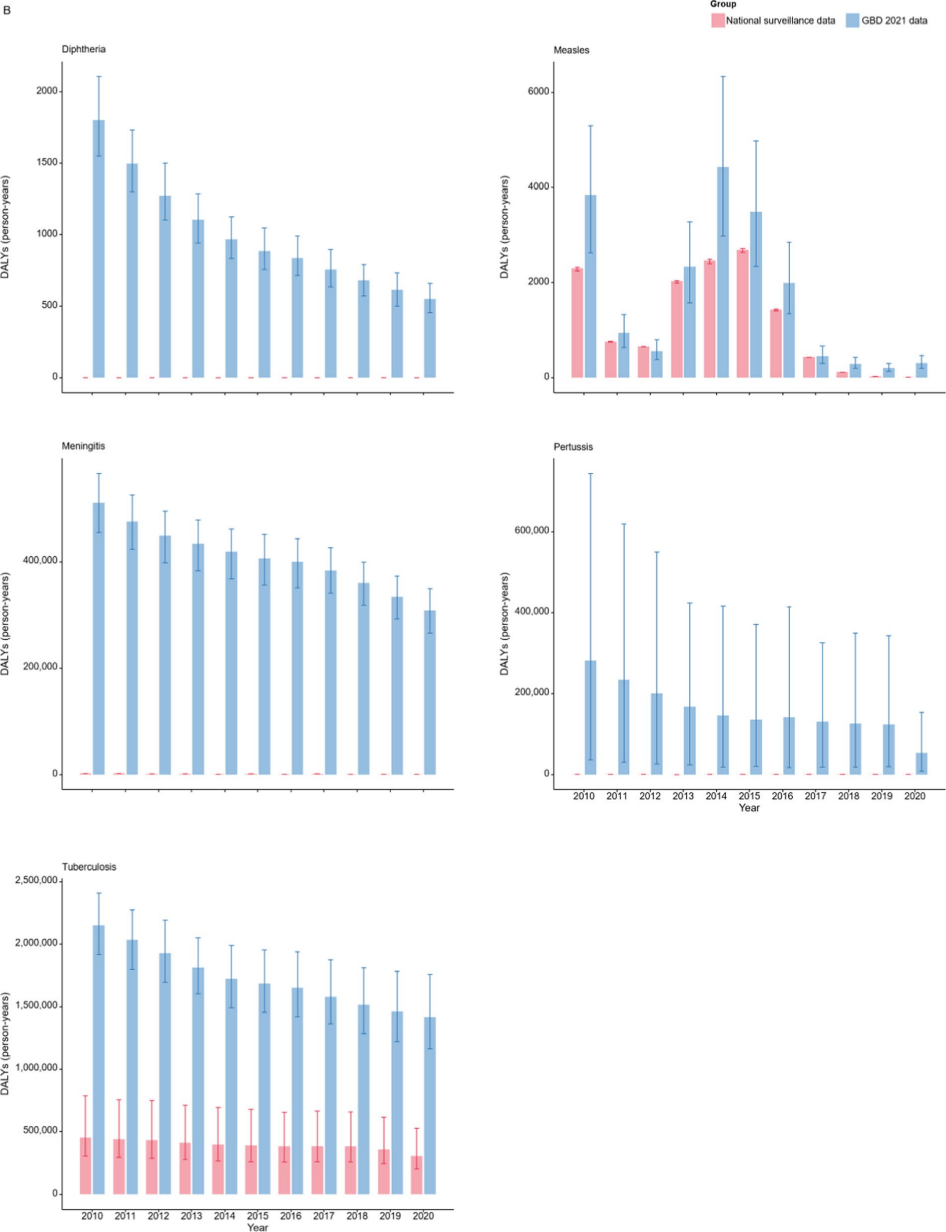

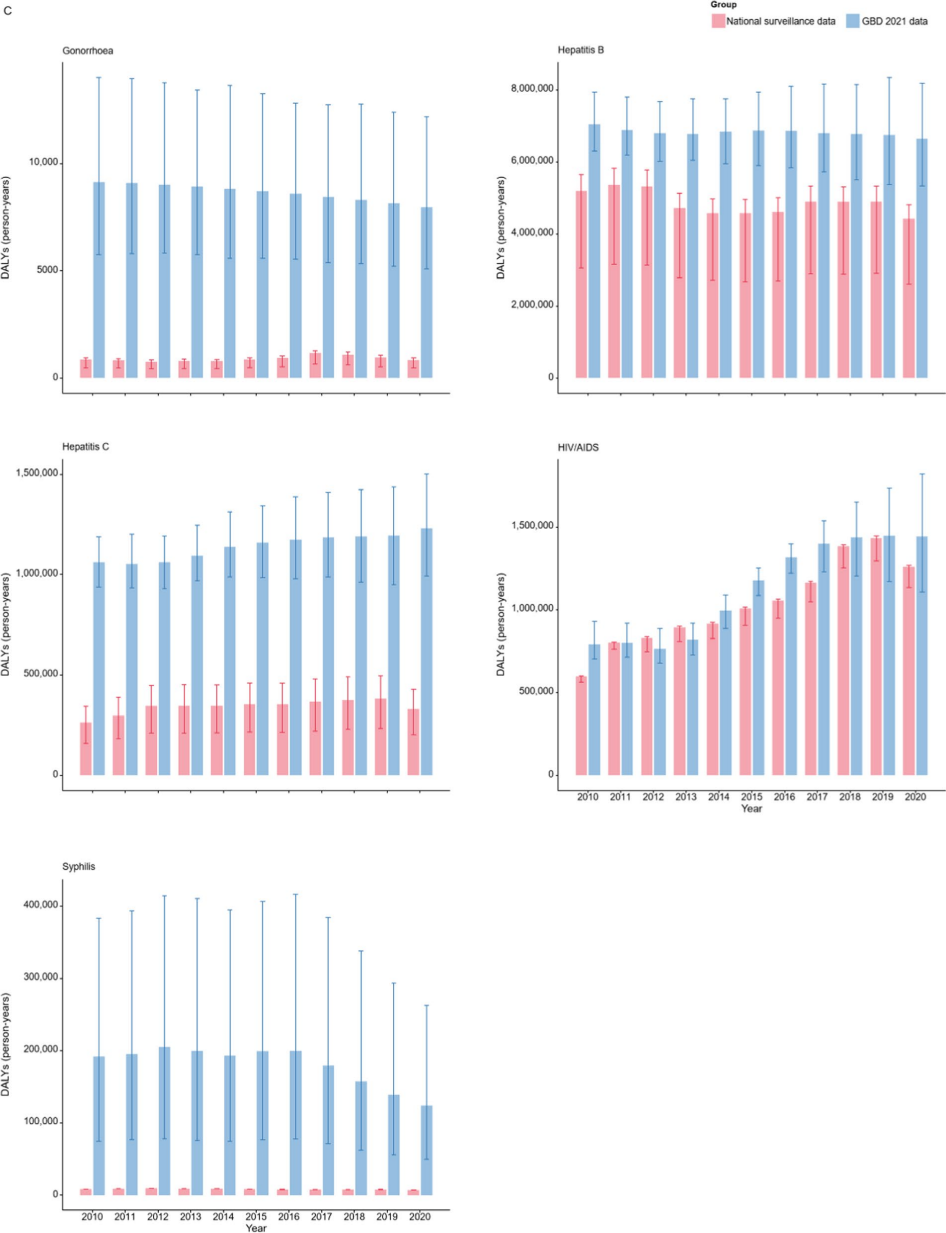
Comparison of Disability-Adjusted Life Years (DALYs) based on national surveillance data with Global Burden of Disease (GBD) 2021 estimates for 14 notifiable infectious diseases (NIDs) in China (2010–2020). **A** Four Intestinal infectious diseases, **B** five respiratory infectious diseases, and **C** five sexually transmitted and blood-borne infections. The bars on the left represent DALYs calculated by national surveillance data, while the bars on the right correspond to GBD 2021 estimates. Error bars indicate the 95% uncertainty intervals of the DALYs

Figure 4 compared the DALYs calculated by reported data of the 14 NIDs with the GBD 2021 estimates. In the case of intestinal infectious diseases (Fig. 4A), the substantial disparity between the GBD 2021 estimates and the calculated DALYs renders the calculated values indiscernible on the same scale. As shown in Fig. 4B and S4 Table, the GBD 2021 model fails to capture the trend of increased disease burden for pertussis during certain periods. Notably, according to GBD 2021, the DALY estimates for diphtheria in China between 2010 and 2019 remained relatively high. However, China did not report any cases of diphtheria or associated deaths during this 10-year period. Figure 4C compares the estimates and calculated DALYs for sexually transmitted and blood-borne infections. Although there is an obvious decrease in the calculated DALYs for hepatitis C and HIV/AIDS in 2020, this reduction is not evident in the GBD 2021 estimates for HIV/AIDS, and even an increase is noted for hepatitis B.

### Infectious diseases prevention and control policies in China

**Fig. 5:**
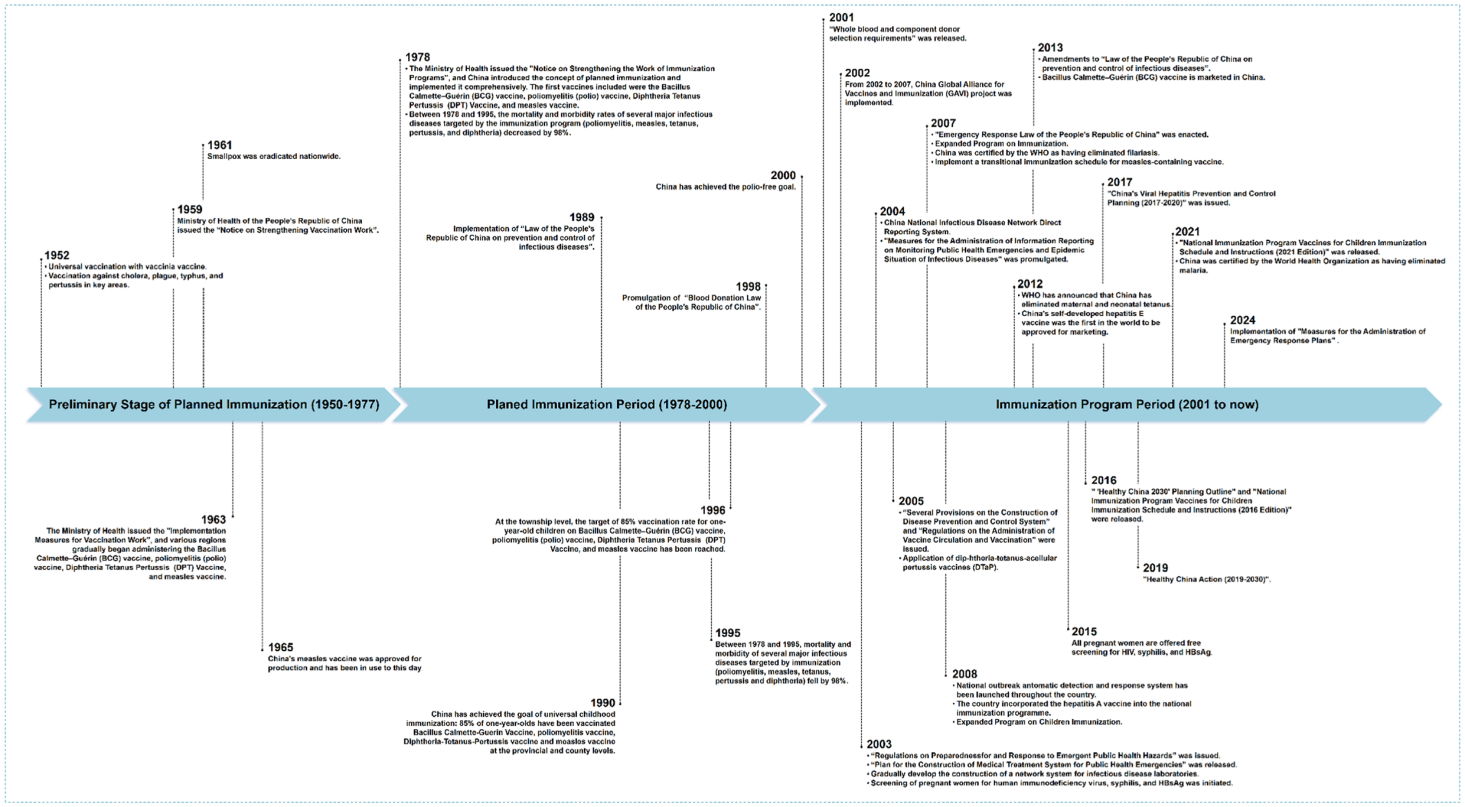
Notifiable infectious diseases (NIDs)-related national policies, guidelines, and milestone accomplishments in China

The control efforts for the infectious diseases in China can be divided into three phases based on the progress of vaccine promotion and coverage: the preliminary stage of planned immunization (1950–1977), the Planned Immunization Period (1978–2000), and the Immunization Planning Period (2000 to now) (Fig. 5). The NIDRIS has enabled real-time online reporting and monitoring of individual cases of NIDs in China, significantly reducing underreporting of cases and deaths for various infectious diseases.

## Discussion

Quantifying disease burden is critical for effective public health planning, especially for infectious diseases that present significant challenges globally and regionally [21]. While the assessment of disease burden continues to evolve, traditional health metrics, such as incidence or mortality rates, have shown limitations in capturing comprehensive disease impact [3]. For example, among the 14 NIDs, despite the relatively lower number of HIV/AIDS cases, its disease burden is significantly high at both the population and individual levels (Fig. 1D). This demonstrates that single-metric approaches may not fully reflect the overall disease burden. The DALYs address this limitation by providing an integrated measure of both mortality and morbidity [22].

While the GBD study has been instrumental in offering global and regional estimates of DALYs, relying solely on GBD model estimates may fail to reflect the realities of specific countries or regions. Our study, based on national surveillance data from China, identified significant discrepancies with GBD 2021 estimates for 14 NIDs, both in rankings and values. GBD 2021 estimates indicate that, from a global perspective, TB (1,725,140.67 person-years) should be prioritized as the second most significant disease for public health interventions in China, followed by hepatitis C (1,141,278.85 person-years), while HIV/AIDS (1,127,936.39 person-years) ranks fourth. In contrast, disease rankings based on national surveillance data suggest that, in China, HIV/AIDS (1,032,155.43 person-years) warrants the second highest level of attention, with TB (394,927.70 person-years), which has a potential comorbid relationship with HIV/AIDS, ranked third (Fig. 3). Such findings highlight the limitations of global models like GBD in capturing the unique epidemiological patterns of specific regions and underscore the importance of data-driven decision-making. Relying solely on global rankings may lead to biased resource allocation. Integrating high-quality, localized monitoring data into disease burden research is essential for formulating context-specific public health strategies, efficiently utilizing resources, and focusing on addressing the most significant health challenges.

The discrepancies between GBD estimates and national surveillance data may be attributed to several factors, many of which are closely linked to China’s specific disease prevention and control strategies. First, GBD’s reliance on multiple data sources, such as national censuses, surveillance systems, and systematic literature reviews, introduces challenges related to data quality and consistency [18, 23]. In contrast, China’s comprehensive legal framework for infectious disease control, supported by the NIDRIS, has significantly enhanced the accuracy and timeliness of disease reporting [24]. Second, GBD is difficult to accurately reflect the impact of long-term immunization strategies. For example, since the inclusion of the Diphtheria-Tetanus-Pertussis vaccine in the National Immunization Program in 1978, the incidence and mortality of diphtheria have dramatically declined, with no reported cases or deaths between 2010 and 2019 [25]. However, GBD still overestimates the burden of diphtheria, failing to capture the success of vaccination efforts (Fig. 4B). Third, the GBD model has inherent limitations in estimating disease burden at the regional level, particularly in accounting for cyclical patterns observed in certain diseases like pertussis (Fig. 4B and S4 Table). Pertussis incidence often fluctuates over time, with periodic peaks and troughs influenced by factors such as waning immunity and vaccination coverage [26]. In contrast, China’s collaborative prevention and control mechanisms, supported by an advanced online reporting network, enable real-time epidemic surveillance. These systems precisely track dynamic changes in disease patterns, providing a more accurate and timely reflection of periodic trends compared to the generalized assumptions used in the GBD model.

To address these discrepancies, this study implemented several methodological optimizations and improvements. First, the GBD model uses a globally standardized reference life table (maximum life expectancy of 86 years), which fails to account for China's unique population structure and rapidly changing mortality patterns [27]. In response, this study utilized data based on China's annual life expectancy, providing a more accurate reflection of the dynamic changes in population health. Second, the severity calibration mechanism of the GBD model applies conservative weights to the disability and mortality risks of infectious diseases, that is, by using higher DWs to ensure that the true burden of disease is not underestimated, which may potentially exaggerate the disease burden [28]. To improve accuracy, this study calculated DWs based on the proportion of symptoms for each infectious disease in China, resulting in more precise burden estimates. These methodological improvements can more directly reflect China's high-quality reported data and avoid uncertainties caused by insufficient data or poor calibration.

However, the purpose of this study is not to negate the GBD methodology but to highlight the value of localized approaches in disease burden assessment. To more accurately capture the true burden of diseases and develop effective response strategies, the WHO has advocated for strengthening national reporting systems [29]. Long before this advocacy, China had already established the world’s largest online reporting network for infectious diseases, covering 168,000 medical institutions and 350,000 users [30]. This system has significantly improved the coverage and quality of disease reporting. In 2005, the nationwide underreporting rate for infectious diseases was 23.14% [31]. Through targeted training programs for healthcare personnel, the underreporting rate decreased to 12.67% by 2008 [32]. By 2009, the total reporting rate had risen to 94.53%, with a timeliness rate of 94.84%, and the underreporting rate further reduced to 5.47% [32]. Currently, the coverage rate of the system in grassroots and above-county-level medical and health institutions is over 94%, and disease prevention and control institutions at or above the county level have achieved full coverage [33]. The average reporting time has been shortened from 5 day to 2 h, with a timeliness rate exceeding 99% [33, 34]. These improvements significantly enhanced the efficiency and quality of infectious disease surveillance and reporting, ensuring the reliability of the data used in this study. For instance, China's success in eliminating malaria is closely linked to the efficient operation of this system [35]. The system also monitors imported cases in real-time, promptly implementing preventive measures to avoid the resurgence of epidemics [31]. In addition, the closed contact tracing system can trace all related pathogens from the index case, ensuring the timely detection and elimination of stage outbreaks of imported epidemics such as dengue [36].

Nonetheless, we acknowledge ongoing challenges in data collection, such as reporting biases and inconsistencies in statistical definitions. To advance research on infectious disease burden, we propose the following recommendations: (1) Continue strengthening disease surveillance and reporting systems; (2) Develop disease burden assessment models tailored to local conditions; (3) Encourage cross-validation using multiple data sources; (4) Establish a multidimensional evaluation framework for infectious disease control to set goals and track progress. Enhancing local surveillance systems, improving global disease burden models, and fostering international collaboration are key steps toward achieving precision in global health strategies and advancing the sustainable development agenda [37].

Several limitations were noted in this study. First, certain diseases were excluded from the analysis due to the lack of available data. Therefore, the findings should be interpreted in the context of the remaining 14 diseases. Second, NIDRIS data relies on nationwide testing and surveillance. If testing resources or monitoring efforts are insufficient in certain regions, it may lead to incomplete or biased data. Third, NIDRIS does not collect case data for different health states across all diseases, which may introduce some bias when determining the DWs and durations. This study addresses this limitation by calculating the 95% uncertainty interval for DALY, although this may complicate the interpretation of disease burden. Significant gender disparities exist in certain infectious diseases due to biological factors (e.g., immune system differences), behaviors (e.g., high-risk activities), and socio-economic or cultural contexts [38]. For instance, men have a higher incidence of tuberculosis, likely due to prolonged indoor exposure to *Mycobacterium tuberculosis* [39]. Studies also show that HIV/AIDS incidence and mortality are higher in men than women in China, highlighting the impact of gender on disease burden [40]. Diseases like hepatitis B also exhibit sex biases, with men generally at higher risk and worse outcomes [41]. However, due to the absence of sex-specific data on incident cases and deaths, this study was unable to calculate sex-specific age-standardized DALYs, which limits the ability to tailor interventions and policies effectively for each gender.

## Conclusions

This study demonstrates considerable discrepancies between the GBD estimates and national surveillance data regarding the burden of 14 NIDs in China, suggesting that relying solely on global datasets such as the GBD may not fully capture the regional or national realities. Therefore, it is essential to strengthen national reporting systems to better reflect the true burden of diseases and develop more effective response strategies. Moreover, promoting the deep integration of the GBD model with localized data and facilitating the development of region-specific models is crucial. While China has made significant breakthroughs in infectious disease prevention and control, substantial challenges remain in controlling and eliminating infectious diseases domestically.

## Supplementary Information

Supplementary Material 1.

## Data Availability

Data analyzed were based on published data. All the original data for the 14 diseases in this study has been made available at https://github.com/Leewudi/China-CDC-14NIDs-raw-data.

## Abbreviations

DALYs: Disability-adjusted life years
GBD: Global Burden of Disease
NIDRIS: National notifiable infectious disease reporting information system
NIDs: Notifiable infectious diseases
CDC: Center for disease control and prevention
SARS: Severe acute respiratory syndrome
H1N1: Influenza A
TB: Tuberculosis
HIV: Human immunodeficiency virus
AIDS: Acquired immunodeficiency syndrome
ICD: International classification of diseases
WHO: World Health Organization
DW: Disability weights
YLLs: Years of life lost
YLDs: Years lived with disability
