# Supplementary material for: Enhancing regional disease burden estimates: insights from the comparison of Global Burden of Disease and China’s notifiable infectious diseases data with policy implications (2010–2020)
Source: Infect Dis Poverty. 2025 Aug 6;14:81. doi: 10.1186/s40249-025-01351-3 (PMC12326785; doi:10.1186/s40249-025-01351-3)
Supplement: Supplementary file 1 — Supplementary Material 1. [file 40249_2025_1351_MOESM1_ESM.docx]

**Supplementary Figures:**

**Figure S1:** Ratios in 11-year average Disability-Adjusted Life Years (DALYs) for 14 notifiable infectious diseases (NIDs) between Global Burden of Disease (GBD) 2021 estimates and national surveillance data in China (2010–2020).

**
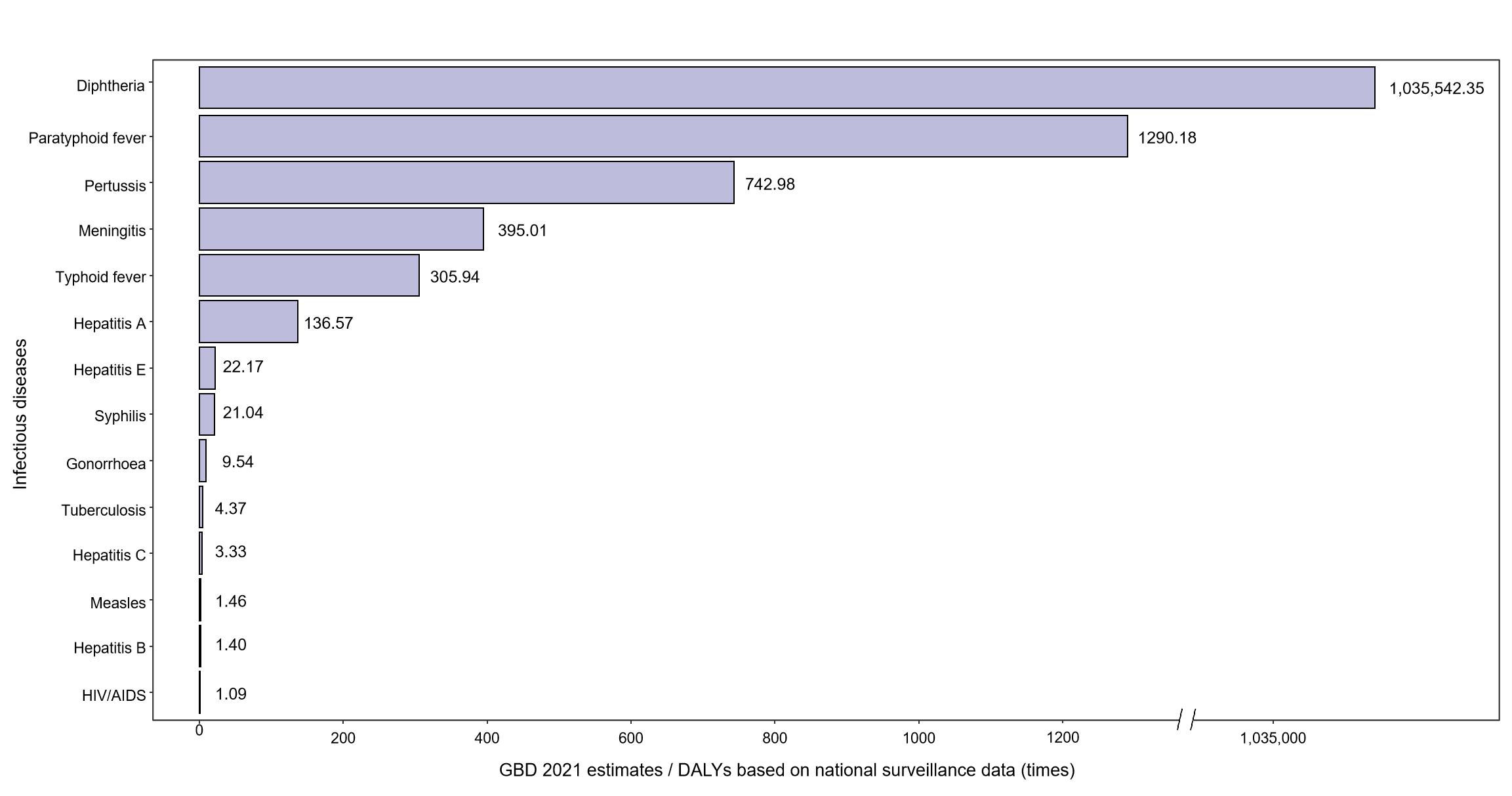
**

**Supplementary Tables:**

**Table S1:** List of International Classification of Diseases (ICD) codes mapped to 14 notifiable infectious diseases (NIDs) in the Global Burden of Disease (GBD) 2021 classification and corresponding codes in China.

| Diseases | GB/T 14396-2001 | GB/T 14396-2016 | ICD-10 |
| --- | --- | --- | --- |
| Hepatitis A | B15 (B15.0-B15.9) | B15.0-B15.9 (B15.000-B15.905) | B15-B15.9 |
| Hepatitis E | B17.2 | B17.2 (B17.200-B17.205) | B17.2 |
| Paratyphoid fever | A01.1-A01.4 | A01.1-A01.4 (A01.100-A01.400) | A01.1-A01.4 |
| Typhoid fever | A01.0 | A01.0 (A01.000) | A01.0-A01.09 |
| Diphtheria | A36 (A36.0-A36.3, A36.8-A36.9) | A36 (A36.0-A36.9) | A36-A36.9 |
| Measles | B05 (B05.0-B05.4, B05.8-B05.9) | B05 (B05.0-B05.9) | B05-B05.9 |
| Meningitis | A39 (A39.06-A39.5, A39.8-A39.9), A87 (A87.0-A87.2, A87.8-A87.9), G00 (G00.0-G00.3, G00.8-G00.9), G01, G02 (G02.0-G02.1, G02.8), G03 (G03.0-G03.2, G03.8-G03.9) | A39 (A39.0-A39.9), A87(A87.0-A87.9), G00 (G00.0-G00.9), G01, G02 (G02.0-G02.1, G02.8), G03 (G03.0-G03.2, G03.8-G03.9) | A39-A39.9, A87-A87.9, D86.81, G00-G03.9, G06-G09.9 |
| Pertussis | A37 (A37.0-A37.1, A37.8-A37.9) | A37 (A37.0-A37.9) | A37-A37.91 |
| Tuberculosis | A15 (A15.0-A15.9), B90 (B90.0-B90.2, B90.8-B90.9), K93.0, M49.0, M90.0, N74.0-N74.1, P37.0, O98.0 | A15-A19 (A15.0-A19.9), B90 (B90.0-B90.9), K93.0, M49.0, M90.0, N74.0-N74.1, P37.0, O98.0 | A10-A14, A15-A18.89, A19-A19.9, B90-B90.9, K67.3, K93.0, M49.0, N74.0-N74.1, P37.0, U84.3 |
| Gonorrhoea | A54 (A54.0-A54.6, A54.8-A54.9), K67.1, M73.0, N74.3, O98.2 | A54 (a54.0-A54.9), K67.1, M73.0, N74.3, O98.2 | A54-A54.9, K67.1, M73.0, N74.3 |
| Hepatitis B | B16 (B16.0-B16.2, B16.9), B17.0, B18.0-B18.1 | B16 (B16.0-B16.2, B16.9), B17.0, B18.0-B18.1 | B16-B16.9, B17.0, B18.0-B18.1, B19.1-B19.11 |
| Hepatitis C | B17.1, B18.2 | B17.1 (B17.000-B17.103), B18.2 (B18.200-B18.205), | B17.1-B17.11, B18.2, B19.2-B19.21 |
| HIV/AIDS | B20-B24, F02.4, Z11.4, Z20.6, Z21, Z83.0 | B20-B24 (B20.000-B24.x01), F02.4, Z11.4, Z20.6, Z21, Z83.0 | B20-B23.8, B24-B24.0, B97.81, C46-C46.52, C46.7-C46.9, F02.4, O98.7-O98.73, Z11.4, Z20.6-Z21, Z83.0 |
| Syphilis | A50-A53, I98.0, K67.2, M73.1, O98.1 | A50-A53 (A50.0-A53.9), I98.0, K67.2, M73.1, O98.1 | A50-A53.9, I98.0, K67.2, M73.1-M73.8 |

Notes: 1. The "Classification and Codes of Diseases" (GB/T 14396-2001) was released on November 2, 2001, and was abolished on February 1, 2017, when it was replaced by the "Classification and Codes of Diseases" (GB/T 14396-2016).

2. GB/T 14396-2016 refines the coding of some diseases based on the GB/T 14396-2001 version. Similarly, the disease coding in both versions in China includes additional refined items based on ICD-10, but after detailed verification, it has been confirmed that these have no impact on the definition and diagnosis of the diseases.

**Table S2:** Disability Weights (DWs) and durations of 14 notifiable infectious diseases (NIDs).

| Diseases | | Symptoms | Disability Weight | DW resource | Duration (year) | Reference |
| --- | --- | --- | --- | --- | --- | --- |
| Hepatitis A | | Low-grade fever; unusual tiredness and weakness; sudden nausea and vomiting and diarrhea; abdominal pain or discomfort; clay- or gray-colored stool; loss of appetite; joint pain; jaundice; intense itching. | 0.058876603  (0.032378223 to 0.189501665) | GBD 2021 | 90/365 | [1,2] |
| Hepatitis E | | Mild fever; loss of appetite; throwing up; belly pain; dark urine and pale or clay-colored stool; skin rash or itching; joint pain; yellowish skin or eyes. | 0.053907609  (0.032378223 to 0.189501665) | GBD 2021 | 56/365 | [3,4] |
| Paratyphoid fever | | Fever; diarrhea; fatigue; headache; loss of appetite; sometimes skin rash. | 0.092003233  (0.032378223 to 0.189501665) | GBD 2021 | 21/365 | [5,6] |
| Typhoid fever | | Fever; headache; chills; loss of appetite; stomach (abdominal) pain; “rose spots” rash, or faint pink spots; cough; muscle aches; nausea, vomiting; diarrhea or constipation. | 0.133411519  (0.088459515 to 0.189501665) | GBD 2021 | 35/365 | [5-8] |
| Diphtheria | | Fever; throat pain; weakness or fatigue; swollen neck glands; dyspnea; dysphagia; nerve, kidney or heart problems (if bacteria enter the bloodstream). | 0.092003233  (0.032378223 to 0.189501665) | GBD 2021 | 21/365 | [9,10] |
| Measles | | High fever; headache; sore throat; barky cough; red or bloodshot eyes; runny nose; red spots with white centers in mouth (Koplik’s spots); rash; digestive symptoms, like diarrhea, stomach pain and vomiting; muscle pain; tiredness. | 0.092003233  (0.032378223 to 0.189501665) | GBD 2021 | 37/365 | [11] |
| Meningitis | | Lack of appetite; nausea or vomiting; sensitivity to light (photophobia); hallucinations; neck stiffness; loss of balance; confusion or altered mental state; lack of attention or focus; lethargy; small round spots that look like a rash (petechiae). | 0.136775938  (0.00419248 to 0.702242795) | GBD 2021 | 37/365 | [5,8,12,13] |
| Pertussis | | Low-grade fever; watery eyes; stuffy nose, runny nose; sore throat; mild or occasional cough that gradually gets worse. | 0.051*  (0.032 to 0.074) | GBD 2019 | 90/365 | [5,14] |
| Tuberculosis | | Persistent cough and fever, shortness of breath, night sweats, weakness and fatigue and severe weight loss. | 0.412831656  (0.223629598 to 0.640202414) | GBD 2021 | 1** | [15,16] |
| Gonorrhoea | | Men have swelling and tenderness in the testicles and pain during urination.  Women have pain in the belly, feels nauseous and have difficulties with daily activities. | 0.097534458  (0.002002058 to 0.44180474) | GBD 2021 | 1/12 | [17,18] |
| Hepatitis B | | Fever; joint pain; anxiety; loss of appetite; swollen belly and swollen legs; nausea and vomiting; stomach pain, abdominal pain; weakness, fatigue; dark urine and light or clay-colored stool; jaundice. | 0.16292396  (0.032378223 to 0.435951652) | GBD 2021 | 30** | [19,20] |
| Hepatitis C | | Fever; body aches; abdominal pain; fatigue; weakness; anxiety; loss of appetite; weight loss; nauseous; swollen belly and swollen legs; jaundice; reddish or dark-colored palms; spider angioma; itchy skin all over; hematochezia or hematemesis; frequent or excessive bleeding and bruising. | 0.170016033  (0.032378223 to 0.435951652) | GBD 2021 | 10** | [14,21] |
| HIV/ AIDS | HIV | Fever; chills; sore throat; mouth ulcers or sores; rash; muscle aches; night sweats; fatigue; swollen lymph nodes; weight loss; frequent infections.  More frequent vaginal yeast infections, missed periods, and other menstrual cycle changes in women.  Urethral discharge, pain or other changes during urination, ulcers or a rash around the penis or anus, rectal discharge or bleeding, pain during bowel movements, and swollen lymph glands in men. | 0.311098216  (0.184358545 to 0.504582025) | GBD 2021 | 6 | [22,23] |
|  | AIDS | Fever that keeps coming back, cough; chills; sweats; ongoing diarrhea; rapid and severe weight loss; constant fatigue; weakness; constant white spots or lesions on the tongue or in the mouth; skin rashes or bumps; swollen lymph glands; frequent infections. | 0.602434504  (0.4055435 to 0.792160094) | GBD 2021 | 5 | [5,23] |
| Syphilis | Congenital syphilis | Fever; aches; weakness; has low intelligence; enlarged liver; jaundice; slight, visible physical deformity; unilateral hearing loss. | 0.056711926  (0.00359251 to 0.28958335) | GBD 2021 | 2 | [17,24] |
|  | Primary syphilis | Low fever and mild discomfort; chancre on the skin or hidden inside the rectum or vagina. | 0.005786497  (0.00229985 to 0.012109072) | GBD 2021 | 45/365 | [17,24] |
|  | Secondary syphilis | Fever; headache; muscle aches; small, reddish-brown sores in mouth, vagina, or anus; swollen glands; weight loss; hair loss; extreme tiredness (fatigue). | 0.005786497  (0.00229985 to 0.012109072) | GBD 2021 | 2 | [17,24] |
|  | Tertiary syphilis | Fever; aches; weakness; stroke; deafness; visual problems or blindness; severe disfigurement, obvious physical deformity; anxiety, personality changes; low intelligence; infection and inflammation of membranes around the brain and spinal cord; neurological problems and cardiovascular complications | 0.343736702  (0.032378223 to 0.690747392) | GBD 2021 | 5** | [17] |
|  | Latent syphilis | Asymptomatic. | 0 | GBD 2021 | - | [17] |

Notes: 1. - indicates that latent syphilis has a DW of 0, and therefore, no disease duration is required.

2. * indicates that the DW values from GBD2019 retain three decimal places.

3. ** indicates that the data were obtained through consultation with infectious disease physicians at the First Affiliated Hospital of Hainan Medical University.

4. The "Reference" column summarizes the references used to determine the proportions of different symptoms and durations for the 14 diseases in China.

**Table S3:** Comparison of Disability-Adjusted Life Years (DALYs) based on national surveillance data and Global Burden of Disease (GBD) 2021 estimates for four intestinal infectious diseases in China from 2004 to 2020.

| Year | Hepatitis A | |  | Hepatitis E | |  | Paratyphoid fever | |  | Typhoid fever | |
| --- | --- | --- | --- | --- | --- | --- | --- | --- | --- | --- | --- |
|  | National surveillance data | GBD 2021 data |  | National surveillance data | GBD 2021 data |  | National surveillance data | GBD 2021 data |  | National surveillance data | GBD 2021 data |
| 2010 | 616.12 | 68,120.32 |  | 731.45 | 12,342.55 |  | 22.89 | 23,121.20 |  | 186.29 | 57,901.27 |
|  | (292.67 to 727.54) | (53,413.90 to 86,146.33) |  | (646.66 to 1107.04) | (8418.55 to 16,420.38) |  | (17.11 to 31.58) | (9384.05 to 47,907.42) |  | (168.35 to 226.31) | (27,170.84 to 10,3624.53) |
| 2011 | 704.96 | 65,352.66 |  | 694.43 | 11,884.23 |  | 16.04 | 21,731.57 |  | 182.50 | 54,922.47 |
|  | (420.02 to 806.27) | (51,176.16 to 84,003.53) |  | (590.21 to 1156.35) | (8153.51 to 16,117.66) |  | (12.03 to 22.08) | (8846.75 to 43,807.75) |  | (166.10 to 218.41) | (25,656.80 to 97,952.19) |
| 2012 | 500.65 | 63,490.61 |  | 616.81 | 11,365.78 |  | 16.84 | 20,299.87 |  | 162.44 | 51,742.91 |
|  | (277.55 to 578.00) | (49,114.49 to 81,164.73) |  | (522.74 to 1047.78) | (7943.44 to 15,477.30) |  | (12.69 to 23.29) | (8300.35 to 41,209.56) |  | (145.83 to 198.72) | (24,535.13 to 90,723.79) |
| 2013 | 375.26 | 62,491.97 |  | 498.05 | 10,851.62 |  | 18.07 | 19,444.26 |  | 229.16 | 50,147.10 |
|  | (171.87 to 446.25) | (47,954.84 to 80,023.43) |  | (399.21 to 938.38) | (7943.44 to 15,477.30) |  | (13.56 to 24.96) | (7892.74 to 39,594.15) |  | (209.34 to 272.99) | (23,499.22 to 88,975.21) |
| 2014 | 488.66 | 61,055.58 |  | 428.50 | 10,441.51 |  | 17.51 | 18,978.41 |  | 133.81 | 49,113.42 |
|  | (251.28 to 572.85) | (46,049.17 to 79,032.16) |  | (334.53 to 851.83) | (7406.13 to 14,821.42) |  | (13.06 to 24.08) | (7774.40 to 38,799.58) |  | (114.17 to 176.83) | (23,374.81 to 87,870.42) |
| 2015 | 620.63 | 60,074.02 |  | 369.27 | 10,206.66 |  | 14.78 | 18,584.49 |  | 126.98 | 47,903.20 |
|  | (415.02 to 693.16) | (45,315.98 to 78,097.50) |  | (273.68 to 795.66) | (7236.89 to 14,381.28) |  | (11.08 to 20.41) | (7643.22 to 37,634.10) |  | (110.34 to 163.72) | (22,724.11 to 86,237.44) |
| 2016 | 370.53 | 59,911.08 |  | 388.18 | 10,295.38 |  | 12.23 | 18,789.77 |  | 153.71 | 47,586.67 |
|  | (175.85 to 437.25) | (45,329.39 to 76,988.73) |  | (294.31 to 832.44) | (7266.72 to 14,973.97) |  | (9.15 to 16.81) | (7751.78 to 38,459.73) |  | (137.65 to 188.99) | (22,495.94 to 86,401.40) |
| 2017 | 325.54 | 58,044.15 |  | 560.25 | 10,381.87 |  | 10.68 | 19,153.86 |  | 184.92 | 47,586.64 |
|  | (156.70 to 385.26) | (43,164.67 to 76,155.17) |  | (459.09 to 1016.98) | (7308.35 to 15,268.91) |  | (8.00 to 14.75) | (7740.34 to 38,737.00) |  | (168.60 to 220.97) | (22,603.21 to 87,088.03) |
| 2018 | 235.13 | 55,437.37 |  | 427.65 | 10,357.53 |  | 14.74 | 19,218.99 |  | 103.10 | 47,229.71 |
|  | (87.05 to 286.36) | (40,039.65 to 73,533.59) |  | (326.24 to 880.06) | (7221.77 to 15,274.81) |  | (11.10 to 20.27) | (7968.88 to 39,437.58) |  | (87.78 to 136.14) | (22,445.02 to 89,542.95) |
| 2019 | 337.45 | 53,490.60 |  | 405.10 | 10,359.54 |  | 11.99 | 18,946.88 |  | 89.65 | 46,498.38 |
|  | (163.27 to 399.43) | (38,997.66 to 71,218.49) |  | (306.89 to 846.80) | (7045.24 to 15,718.49) |  | (8.91 to 16.51) | (7756.48 to 37,888.54) |  | (76.59 to 118.54) | (21,765.93 to 84,151.15) |
| 2020 | 255.94 | 52,274.44 |  | 341.73 | 10,444.54 |  | 8.70 | 13,939.26 |  | 205.82 | 37,317.70 |
|  | (121.14 to 303.22) | (37,355.38 to 70,055.26) |  | (275.33 to 640.19) | (7163.71 to 15,625.47) |  | (6.52 to 12.01) | (5677.32 to 28,416.97) |  | (196.05 to 227.96) | (17,124.87 to 65,113.08) |

Notes: The value for each disease is presented as DALYs (person-years).

**Table S4:** Comparison of Disability-Adjusted Life Years (DALYs) based on national surveillance data and Global Burden of Disease (GBD) 2021 estimates for five respiratory infectious diseases in China from 2004 to 2020.

| Year | Diphtheria | |  | Measles | |  | Meningitis | |  | Pertussis | |  | Tuberculosis | |
| --- | --- | --- | --- | --- | --- | --- | --- | --- | --- | --- | --- | --- | --- | --- |
|  | National surveillance data | GBD 2021 data |  | National surveillance data | GBD 2021 data |  | National surveillance data | GBD 2021 data |  | National surveillance data | GBD 2021 data |  | National surveillance  data | GBD 2021 data |
| 2010 | 0.00 | 1800.89 |  | 2296.79 | 3839.12 |  | 2004.40 | 51,1366.21 |  | 96.51 | 281,395.97 |  | 454,074.66 | 2,152,567.16 |
|  | (0.00 to 0.00) | (1550.73 to 2105.54) |  | (2241.93 to 2317.77) | (2623.38 to 5302.15) |  | (2003.70 to 2019.09) | (455,588.85 to 566,267.67) |  | (94.26 to 103.50) | (35,926.99 to 744,696.38) |  | (304,564.48 to 790,641.09) | (1,916,773.86 to 2,406,995.74) |
| 2011 | 0.00 | 1496.80 |  | 757.03 | 938.42 |  | 1672.41 | 475,822.62 |  | 179.31 | 235,011.48 |  | 438,943.93 | 2,034,808.81 |
|  | (0.00 to 0.00) | (1299.84 to 1732.69) |  | (742.77 to 762.33) | (636.97 to 1324.97) |  | (1671.93 to 1682.80) | (423,778.37 to 525,717.39) |  | (176.11 to 189.22) | (29,595.93 to 619,451.02) |  | (296,664.95 to 757,157.20) | (1,800,957.72 to 2,274,481.18) |
| 2012 | 0.00 | 1272.15 |  | 651.30 | 559.42 |  | 1507.62 | 449,027.48 |  | 100.78 | 200,731.87 |  | 433,126.83 | 1,930,475.29 |
|  | (0.00 to 0.00) | (1102.39 to 1499.92) |  | (642.59 to 654.66) | (377.56 to 799.00) |  | (1507.17 to 1516.57) | (398,909.72 to 495,230.24) |  | (98.06 to 109.49) | (25,152.90 to 549,842.71) |  | (290,329.70 to 749,600.76) | (1,693,546.27 to 2,191,518.74) |
| 2013 | 0.00 | 1102.55 |  | 2030.76 | 2335.69 |  | 1310.88 | 433,955.15 |  | 21.53 | 168,331.12 |  | 412,602.80 | 1,813,094.53 |
|  | (0.00 to 0.00) | (940.29 to 1285.74) |  | (1990.99 to 2045.57) | (1570.31 to 3270.48) |  | (1310.43 to 1320.59) | (383,755.27 to 479,241.61) |  | (19.37 to 28.40) | (23,388.19 to 424,494.14) |  | (277,767.46 to 713,891.51) | (1,604,483.85 to 2,050,178.44) |
| 2014 | 0.00 | 968.39 |  | 2467.07 | 4430.89 |  | 764.32 | 419,305.67 |  | 191.52 | 145,898.24 |  | 400,404.00 | 1,724,611.28 |
|  | (0.00 to 0.00) | (832.92 to 1124.64) |  | (2391.07 to 2495.52) | (2978.35 to 6329.26) |  | (763.94 to 772.05) | (368,073.94 to 461,712.88) |  | (187.26 to 204.91) | (18,224.32 to 416,940.62) |  | (267,081.55 to 696,868.71) | (1,493,922.86 to 1,990,770.23) |
| 2015 | 0.00 | 887.00 |  | 2692.63 | 3485.85 |  | 878.39 | 406,796.81 |  | 235.41 | 136,043.41 |  | 391,627.14 | 1,683,913.23 |
|  | (0.00 to 0.00) | (755.27 to 1046.64) |  | (2631.80 to 2715.46) | (2335.61 to 4981.51) |  | (878.15 to 883.15) | (356,896.72 to 451,617.00) |  | (227.01 to 262.03) | (20,537.06 to 371,027.88) |  | (262,324.15 to 680,822.30) | (1,455,959.29 to 1,954,992.84) |
| 2016 | 0.00 | 836.64 |  | 1431.60 | 1987.29 |  | 682.80 | 399,791.77 |  | 297.74 | 140,887.76 |  | 381,716.85 | 1,652,511.91 |
|  | (0.00 to 0.00) | (715.85 to 991.21) |  | (1396.07 to 1444.81) | (1347.49 to 2849.23) |  | (682.60 to 687.39) | (351,103.17 to 443,988.40) |  | (290.67 to 320.07) | (17,610.38 to 414,344.53) |  | (257,462.41 to 656,115.35) | (1,422,795.31 to 1,940,297.25) |
| 2017 | 0.00 | 754.78 |  | 429.61 | 454.68 |  | 1206.60 | 383,266.93 |  | 130.66 | 130,503.05 |  | 384,910.75 | 1,582,251.37 |
|  | (0.00 to 0.00) | (635.90 to 894.92) |  | (421.04 to 432.77) | (303.43 to 663.80) |  | (1206.35 to 1211.96) | (340,707.08 to 426,146.23) |  | (117.51 to 172.25) | (18,581.00 to 325,859.39) |  | (261,988.76 to 664,143.72) | (1,360,672.97 to 1,875,892.00) |
| 2018 | 0.00 | 678.97 |  | 112.59 | 286.92 |  | 682.84 | 360,810.58 |  | 429.05 | 125,377.74 |  | 383,565.92 | 1,518,277.59 |
|  | (0.00 to 0.00) | (571.60 to 790.43) |  | (106.90 to 114.74) | (190.22 to 424.73) |  | (682.62 to 687.57) | (318,595.43 to 399,397.14) |  | (402.18 to 516.55) | (18,494.88 to 349,307.28) |  | (257,126.44 to 659,775.33) | (1,284,462.70 to 1,813,522.41) |
| 2019 | 0.00 | 613.63 |  | 27.74 | 204.22 |  | 428.58 | 334,240.91 |  | 529.28 | 124,511.86 |  | 358,873.50 | 1,463,417.22 |
|  | (0.00 to 0.00) | (499.06 to 732.49) |  | (23.50 to 29.35) | (135.26 to 299.78) |  | (428.33 to 433.62) | (292,199.93 to 373,100.62) |  | (491.72 to 649.90) | (19,092.56 to 343,395.04) |  | (244,752.76 to 618,698.81) | (1,219,564.63 to 1,782,683.04) |
| 2020 | 0.01 | 551.15 |  | 7.98 | 307.46 |  | 210.98 | 308,895.76 |  | 133.70 | 53,962.09 |  | 304,358.26 | 1,420,618.97 |
|  | (0.01 to 0.02) | (455.27 to 659.01) |  | (6.76 to 8.46) | (192.61 to 468.10) |  | (210.87 to 213.26) | (265,903.25 to 349,532.62) |  | (128.10 to 151.53) | (7863.76 to 153,447.92) |  | (203,919.61 to 527,034.50) | (1,164,507.27 to 1,759,225.15) |

Notes: The value for each disease is presented as DALYs (person-years).

**Table S5:** Comparison of Disability-Adjusted Life Years (DALYs) based on national surveillance data and Global Burden of Disease (GBD) 2021 estimates for five sexually transmitted and blood-borne infections in China from 2004 to 2020.

| Year | Gonorrhoea | |  | Hepatitis B | |  | Hepatitis C | |  | HIV/AIDS | |  | Syphilis | |
| --- | --- | --- | --- | --- | --- | --- | --- | --- | --- | --- | --- | --- | --- | --- |
|  | National surveillance data | GBD 2021 data |  | National surveillance  data | GBD 2021 data |  | National surveillance  data | GBD 2021 data |  | National surveillance  data | GBD 2021 data |  | National surveillance data | GBD 2021 data |
| 2010 | 860.18 | 9140.81 |  | 5,199,987.47 | 7,040,421.46 |  | 262,777.82 | 1,061,481.29 |  | 597,226.99 | 793,148.56 |  | 8243.05 | 191,957.11 |
|  | (476.15 to 944.67) | (5752.12 to 14,013.80) |  | (3,058,581.56 to 5,657,964.05) | (6,311,982.60 to 7,944,232.27) |  | (160,320.07 to 343,259.44) | (938,651.13 to 1,190,549.87) |  | (565,123.00 to 599,759.80) | (701,071.48 to 930,811.13) |  | (8313.54 to 8413.20) | (74,661.54 to 383,266.90) |
| 2011 | 828.49 | 9094.88 |  | 5,358,857.05 | 6,895,373.93 |  | 298,179.25 | 1,053,445.11 |  | 804,464.52 | 801,716.29 |  | 8781.10 | 195,526.35 |
|  | (472.55 to 905.25) | (5804.36 to 13,960.25) |  | (3,164,633.29 to 5,831,691.54) | (6,187,802.56 to 7,806,417.35) |  | (182,135.55 to 387,906.97) | (934,384.56 to 1,201,965.78) |  | (763,281.38 to 807,728.93) | (715,925.55 to 919,239.04) |  | (8861.31 to 8970.92) | (76,940.15 to 393,910.34) |
| 2012 | 773.90 | 9022.87 |  | 5,325,433.93 | 6,805,230.03 |  | 345,298.07 | 1,062,369.12 |  | 832,438.52 | 765,415.48 |  | 9476.77 | 205,285.61 |
|  | (439.74 to 847.43) | (5817.81 to 13,759.74) |  | (3,137,618.07 to 5,775,464.18) | (6,009,642.26 to 7,667,940.83) |  | (209,531.36 to 449,671.90) | (930,530.39 to 1,192,864.45) |  | (747,556.59 to 839,115.26) | (678,956.88 to 889,063.38) |  | (9564.26 to 9676.95) | (78,472.46 to 414,686.98) |
| 2013 | 810.02 | 8926.26 |  | 4,719,195.12 | 6,789,099.55 |  | 348,364.62 | 1,096,137.69 |  | 893,164.84 | 821,032.07 |  | 8611.94 | 199,675.72 |
|  | (456.81 to 886.06) | (5753.98 to 13,426.98) |  | (2,791,079.27 to 5,131,367.76) | (6,045,776.63 to 7,750,969.08) |  | (210,140.40 to 453,247.16) | (969,099.89 to 1,246,796.77) |  | (808,464.67 to 899,753.23) | (727,489.69 to 918,050.09) |  | (8702.86 to 8815.79) | (75,924.76 to 410,550.33) |
| 2014 | 795.65 | 8816.08 |  | 4,581,108.18 | 6,841,511.01 |  | 347,824.93 | 1,140,316.06 |  | 916,763.91 | 995,305.09 |  | 8706.87 | 193,475.39 |
|  | (450.51 to 870.00) | (5595.50 to 13,647.70) |  | (2,717,842.31 to 4,976,721.25) | (5,958,635.52 to 7,759,184.43) |  | (212,390.81 to 451,045.27) | (987,654.24 to 1,313,359.65) |  | (827,149.95 to 923,901.52) | (888,809.27 to 1,087,963.39) |  | (8811.24 to 8926.59) | (74,634.83 to 395,270.02) |
| 2015 | 858.62 | 8705.62 |  | 4,574,352.54 | 6,873,600.61 |  | 355,536.27 | 1,159,633.86 |  | 1,007,071.48 | 1,178,703.61 |  | 8130.38 | 199,438.68 |
|  | (497.39 to 938.55) | (5580.94 to 13,251.47) |  | (2,680,448.88 to 4,964,646.49) | (5,894,358.29 to 7,941,342.69) |  | (215,633.59 to 461,619.36) | (986,271.65 to 1,342,524.60) |  | (905,595.89 to 1,015,341.57) | (1,086,916.00 to 1,253,108.05) |  | (8249.27 to 8368.45) | (76,681.94 to 40,6859.28) |
| 2016 | 948.74 | 8609.45 |  | 4,614,011.10 | 6,870,298.38 |  | 353,935.16 | 1,175,489.90 |  | 1,057,220.56 | 1,317,021.81 |  | 7758.78 | 19,9617.89 |
|  | (530.67 to 1038.07) | (5542.04 to 12,822.45) |  | (2,705,208.01 to 5,005,587.17) | (5,844,268.83 to 8,104,544.58) |  | (213,812.21 to 460,491.16) | (979,476.76 to 1,388,767.83) |  | (947,918.17 to 1,065,554.80) | (1,221,451.45 to 1,399,233.41) |  | (7888.53 to 8008.90) | (77,680.96 to 416,684.47) |
| 2017 | 1172.44 | 8440.69 |  | 4,905,822.75 | 6,811,803.36 |  | 366,287.77 | 1,185,468.50 |  | 1,164,692.10 | 1,400,723.72 |  | 7477.23 | 179,574.54 |
|  | (669.70 to 1279.50) | (5384.86 to 12,735.44) |  | (2,902,305.92 to 5,323,730.95) | (5,722,527.52 to 8,163,874.16) |  | (221,016.40 to 479,661.69) | (988,946.43 to 1,410,106.18) |  | (1,049,713.12 to 1,173,596.60) | (1,228,355.81 to 1,540,742.79) |  | (7622.86 to 7754.12) | (71,118.35 to 384,712.18) |
| 2018 | 1111.11 | 8313.74 |  | 4,895,816.29 | 6,779,563.13 |  | 374,682.67 | 1,191,505.09 |  | 1,384,774.24 | 1,438,886.63 |  | 7580.01 | 157,189.95 |
|  | (630.10 to 1215.43) | (5340.37 to 12,763.69) |  | (2,884,706.09 to 5,318,108.00) | (5,516,375.50 to 8,146,279.69) |  | (229,826.89 to 489,921.36) | (962,939.02 to 1,425,739.25) |  | (1,254,766.03 to 1,395,332.67) | (1,206,521.32 to 1,652,452.30) |  | (7736.59 to 7873.72) | (62,444.70 to 338,172.52) |
| 2019 | 958.58 | 8161.00 |  | 4,908,186.50 | 6,744,653.69 |  | 382,218.94 | 1,196,134.78 |  | 1,436,555.58 | 1,448,766.69 |  | 7702.27 | 139,412.45 |
|  | (529.92 to 1053.49) | (5222.35 to 12,390.18) |  | (2,913,667.11 to 5,330,029.54) | (5,375,468.46 to 8,351,332.35) |  | (232,928.65 to 496,855.74) | (951,438.94 to 1,437,821.01) |  | (1,294,351.10 to 1,447,385.91) | (1,170,610.13 to 1,736,724.52) |  | (7878.96 to 8027.62) | (56,123.74 to 293,781.87) |
| 2020 | 854.73 | 7952.40 |  | 4,421,540.26 | 6,645,349.71 |  | 332,155.88 | 1,232,085.99 |  | 1,259,336.98 | 1,446,580.33 |  | 7086.21 | 124,288.59 |
|  | (474.56 to 937.57) | (5100.43 to 12,160.50) |  | (2,600,897.11 to 4,808,589.42) | (5,336,235.65 to 8,188,833.85) |  | (201,367.42 to 430,300.24) | (994,539.86 to 1,501,494.25) |  | (1,134,771.53 to 1,269,389.65) | (1,107,380.15 to 1,820,987.03) |  | (7244.51 to 7371.86) | (49,926.17 to 262,803.63) |

Notes: The value for each disease is presented as DALYs (person-years).

**Reference:**

1. Shanghai Municipal Center for Disease Control and Prevention. Diagnostic criteria of viral hepatitis A. Ministry of Health of the People's Republic of China; 2008. <https://icdc.chinacdc.cn/zcfgybz/bz/202112/P020211202493027020948.pdf>. Accessed 28 Sept 2024.

2. World Health Organization. Hepatitis A. Geneva: World Health Organization; 2023. <https://www.who.int/news-room/fact-sheets/detail/hepatitis-a>. Accessed 28 Sept 2024.

3. World Health Organization. Hepatitis E. Geneva: World Health Organization; 2023. <https://www.who.int/news-room/fact-sheets/detail/hepatitis-e>. Accessed 27 Sept 2024.

4. Chinese Consortium for the Study of Hepatitis E (CCSHE), Chinese Physician Association for Infectious Disease, National Clinical Research Center for Infectious Diseases. Expert consensus on the process of in-hospital screening and management of viral hepatitis E in China (2023). Chin J Lab Med. 2023;46(6):558-65. (in Chinese).

5. Li LJ, Ren H. Infectious Diseases. 9th ed. Beijing: People's Medical Publishing House; 2018.

6. National Disease Control and Prevention Administration. Typhoid fever and paratyphoid fever. National Disease Control and Prevention Administration; 2023. <https://www.ndcpa.gov.cn/jbkzzx/c1000372/common/content/content_1715236259037048832.html>. Accessed 19 Sept 2024.

7. Bush LM, Vazquez-Pertejo MT. Typhoid fever. MSD Manuals; 2022. <https://www.msdmanuals.cn/professional/infectious-diseases/gram-negative-bacilli/typhoid-fever>. Accessed 28 Sept 2024.

8. Liang WN. Identification and treatment of notifiable infectious diseases. Beijing: Peking Union Medical College Press; 2005.

9. Chinese Center for Disease Control and Prevention. Diphtheria. Chinese Center for Disease Control and Prevention; 2024. <https://www.chinacdc.cn/jkyj/mygh02/jbzt/xjxcrb/bh/>. Accessed 26 Sept 2024.

10. Heilongjiang Provincial Center for Disease Control and Prevention, Chinese Center for Disease Control and Prevention, First Affiliated Hospital of Harbin Medical University. Diagnostic criteria for diphteria. Ministry of Health of the People's Republic of China; 2007. <https://icdc.chinacdc.cn/zcfgybz/bz/202112/P020211202500995128043.pdf>. Accessed 26 Sept, 2024.

11. General Office of the National Health Commission, Comprehensive Department of the State Administration of Traditional Chinese Medicine. Diagnosis and treatment plan for measles (2024 version). Chin J Viral Dis. 2024;14(05):401-5. 10.16505/j.2095-0136.2024.0073.

12. National Health Commission of the People’s Republic of China. Diagnosis and Treatment Plan for Epidemic Cerebrospinal Meningitis (2023 Edition). Chinese Practical Journal of Rural Doctor. 2024;31(06):6-9.

13. World Health Organization. Meningitis. Geneva: World Health Organization; 2023. <https://www.who.int/news-room/fact-sheets/detail/meningitis>. Accessed 27 Sept 2024.

14. Chinese Society of Hepatology, Chinese Medical Association, Chinese Society of Infectious Diseases, Chinese Medical Association. Guideline for the prevention and treatment of hepatitis C (2022 version). Chinese Journal of Clinical Infectious Diseases. 2022;428-447(06):428-47. (in Chinese).

15. Edward AN. Tuberculosis(TB). MSD Manuals; 2022. <https://www.msdmanuals.cn/home/infections/tuberculosis-and-related-infections/tuberculosis-tb>. Accessed 24 Sept 2024.

16. World Health Organization. Tuberculosis. Geneva: World Health Organization; 2023. <https://www.who.int/zh/news-room/fact-sheets/detail/tuberculosis>. Accessed 24 Sept 2024.

17. National Center for STD Control, Chinese Center for Disease Control and Prevention, Venereology Group, Chinese Society of Dermatology, Subcommittee on Venereology, China Dermatologist Association. Guidelines for diagnosis and treatment of syphilis, gonorrhea and genital Chlamydia trachomatis infection (2020). Chin J Dermatol. 2020;53(03):168-79. (in Chinese).

18. World Health Organization. Gonorrhoea (Neisseria gonorrhoeae infection). Geneva: World Health Organization; 2024. <https://www.who.int/news-room/fact-sheets/detail/gonorrhoea-(neisseria-gonorrhoeae-infection>). Accessed 16 Nov 2024.

19. World Health Organization. Hepatitis B. Geneva: World Health Organization; 2024. <https://www.who.int/news-room/fact-sheets/detail/hepatitis-b>. Accessed 24 Sept 2024.

20. Chinese Society of Hepatology, Chinese Medical Association, Chinese Society of Infectious Diseases, Chinese Medical Association. Guidelines for the prevention and treatment of chronic hepatitis B (version 2022). Chinese Journal of Hepatology. 2022;30(12):1309-31.

21. Chinese Center for Disease Control and Prevention. Current status of hepatitis C case reporting in China. Chinese Center for Disease Control and Prevention; 2019. <https://ncaids.chinacdc.cn/sjb/zsyd_10323/201906/t20190605_203086.htm>. Accessed 19 Sept 2024.

22. Acquired Immunodeficiency Syndrome Professional Group SOID, Chinese Medical Association;, Prevention CCFDCA. Chinese guidelines for diagnosis and treatment of human immunodeficiency virus infection/acquired immunodeficiency syndrome (2024 edition). Chinese Journal of Infectious Diseases. 2024;42(05):257-84. (in Chinese).

23. World Health Organization. HIV and AIDS. Geneva: World Health Organization; 2023. <https://www.who.int/news-room/fact-sheets/detail/hiv-aids>. Accessed 22 Sept 2024.

24. World Health Organization. Syphilis. Geneva: World Health Organization; 2024. <https://www.who.int/news-room/fact-sheets/detail/syphilis>. Accessed 22 Sept 2024.
